# Burnout among public health workers in Canada: a cross-sectional study

**DOI:** 10.1186/s12889-023-17572-w

**Published:** 2024-01-02

**Authors:** Japteg Singh, David E-O Poon, Elizabeth Alvarez, Laura Anderson, Chris P. Verschoor, Arielle Sutton, Zayya Zendo, Thomas Piggott, Emma Apatu, Donna Churipuy, Ian Culbert, Jessica P. Hopkins

**Affiliations:** 1 Niagara Region Public Health, Thorold, ON Canada; 2 Public Health and Preventive Medicine Residency Program, Dalla Lana School of Public Health, University of Toronto, Toronto, ON Canada; 3 Department of Health Research Methods, Evidence, and Impact, McMaster University, Hamilton, ON Canada; 4 Health Sciences North Research Institute, Sudbury, ON Canada; 5 MD Program, Faculty of Medicine and Dentistry, Schulich School of Medicine, Western University, London, ON Canada; 6 Peterborough Public Health, Peterborough, ON Canada; 7 Canadian Public Health Association, Ottawa, ON Canada; 8 Public Health Ontario, Toronto, ON Canada

**Keywords:** Burnout, Canadian public health workforce, Oldenburg burnout inventory, Pandemic, Workforce planning

## Abstract

**Background:**

This study presents the prevalence of burnout among the Canadian public health workforce after three years of the COVID-19 pandemic and its association with work-related factors.

**Methods:**

Data were collected using an online survey distributed through Canadian public health associations and professional networks between November 2022 and January 2023. Burnout was measured using a modified version of the Oldenburg Burnout Inventory (OLBI). Logistic regressions were used to model the relationship between burnout and work-related factors including years of work experience, redeployment to pandemic response, workplace safety and supports, and harassment. Burnout and the intention to leave or retire as a result of the COVID-19 pandemic was explored using multinomial logistic regressions.

**Results:**

In 2,079 participants who completed the OLBI, the prevalence of burnout was 78.7%. Additionally, 49.1% of participants reported being harassed because of their work during the pandemic. Burnout was positively associated with years of work experience, redeployment to the pandemic response, being harassed during the pandemic, feeling unsafe in the workplace and not being offered workplace supports. Furthermore, burnout was associated with greater odds of intending to leave public health or retire earlier than anticipated.

**Conclusion:**

The high levels of burnout among our large sample of Canadian public health workers and its association with work-related factors suggest that public health organizations should consider interventions that mitigate burnout and promote recovery.

**Supplementary Information:**

The online version contains supplementary material available at 10.1186/s12889-023-17572-w.

## Background

The coronavirus disease 2019 (COVID-19) pandemic has had significant health and social impacts globally. The first case of COVID-19 in Canada was identified in January 2020, and there have been more than 4.6 million cases and 52,000 deaths in the country as of June 13, 2023 [1]. In Canada, responsibility for delivering public health programs and services to the population is shared between federal, provincial and regional/local public health agencies. Across Canada, public health authorities provide service to all people in Canada with local/regional health authorities focusing on local communities, provincial/territorial authorities focusing on coordination and overarching services in provinces/territories, and federal authorities coordinating work across Canada, as well as specifically providing services to First Nations and Inuit communities and armed forces. Throughout the course of the pandemic, public health workers have been responsible for planning, implementing and evaluating the COVID-19 response, as well as delivering other essential public health services that could not be deferred (e.g., high-risk child and family programs, harm reduction services). The magnitude of the response required many public health workers to be redeployed from their usual program areas and positions. While Canada’s public health response to COVID-19 has evolved, strategies for minimizing the spread of the disease have included border measures and travel restrictions; case, contact and outbreak management; testing and laboratory services; stay-at-home orders and closure of non-essential services; public education; masking and vaccination programs [2]. Given the intensity and length of the pandemic response, there has been growing concern over the risk of burnout among the public health workforce in Canada.

Burnout is recognized as a major occupational health syndrome characterized by emotional exhaustion, depersonalization and diminished sense of achievement from the chronic exposure to stressors in the workplace [3]. Burnout may lead to poor mental health outcomes including anxiety, depression, fatigue and suicidal ideation [4, 5]. It may also have organizational consequences such as absenteeism, job dissatisfaction, interpersonal strain, reduced job performance and, in healthcare settings, poor quality of care or impaired patient safety [6]. Since the pandemic, several studies have described levels of burnout among Canadian healthcare providers. Among physicians, the prevalence of burnout during the pandemic increased from 30% in 2018 [7] to upwards of 68–86% in 2021 [8, 9]. In nurses, 75% were found to have burnout [10].

As a result of the COVID-19 pandemic, there has been increased interest in exploring workforce burnout, however, few studies have attempted to explore burnout among the Canadian public health workforce. In jurisdictions outside of Canada, burnout rates among public health workforces during the pandemic were 45% in Malaysia [11], 66% in the United States of America (USA) [12], and 90% in South Korea [13], and pre-pandemic 50% in China [14]. We sought to measure the prevalence of burnout in the Canadian public health workforce three years into the COVID-19 pandemic, and to explore any associated work-related factors. In addition, we explored burnout and the intention to leave public health or retire early during the pandemic.

## Methods

Reporting of this study followed the Checklist for Reporting Results of Internet E-Surveys (CHERRIES) [15] and the Strengthening the Reporting of Observational Studies in Epidemiology (STROBE) reporting guidelines for observational studies [16].

### Study design and participants

In this cross-sectional study, a convenience sample of Canadian public health practitioners were invited to participate in an online survey from November 22, 2022 to January 17, 2023. A convenience sample using broad outreach across national, provincial/territorial and local professional associations and networks was used as there is no census, enumeration or listing of public health workers in the country. Participation in the study was voluntary, survey responses were anonymized and reported in aggregate. Persons 18 years and older who self-identified as a public health practitioner and worked any time between December 2019 and January 2023 were eligible. A public health practitioner was defined as a person whose work aligned with one or more of the following core functions for Public Health in Canada: population health assessment, health protection, health surveillance, disease and injury prevention, health promotion and/ or emergency preparedness and response [17]. This ensured our study sample reflected the diversity of professions in the public health field as well as restricted responses to those active during the COVID-19 pandemic response. Practicum students and individuals working outside of Canada were excluded from the study.

The survey was available in English and French through Surveys@PHO, a password protected web-based application at Public Health Ontario, which is designed for survey development and data management. Invitation emails were distributed through Canadian local, provincial and national public health associations and professional groups (Appendix A).

For the primary aim of measuring the prevalence of burnout, a minimum sample size was calculated *a priori* using the following equation: *N = Z*^*2*^*P*(1 − *P*)/*d*^*2*^. Assuming a confidence level (Z) of 95%, margin of error (d) of 5% and estimated prevalence (P) of 50% to maximize the sample size calculation, a sample of at least 384 participants was deemed necessary.

### Study measures

The questionnaire consisted of three parts and collected information regarding demographic factors, work related-factors, and burnout using closed and open-ended questions (Appendix B). In this study, we explored the following work-related factors in relation to burnout: years of work experience, redeployment to the pandemic response, length of redeployment, threatened, assaulted or bullied during the pandemic, feeling safe in the workplace, offered workplace supports for physical and/or mental wellbeing and intention to leave or retire as a result of the pandemic.

Burnout was measured using a modified version of the validated Oldenburg Burnout Inventory (OLBI), a 16-item Likert-type questionnaire consisting of two subscales: exhaustion and disengagement [18]. Exhaustion refers to general feelings of emptiness, work overload, strong need for rest and cognitive, emotional and physical exhaustion [3]. Disengagement relates to feelings of withdrawal from work and negative attitudes and behaviours towards work [3]. Each burnout subscale consisted of eight items: four positively-worded and four negatively-worded statements presented in mixed order with responses constructed on a 4-point Likert-scale ranging from totally disagree to totally agree. The item, *“After work, I tend to need more time than in the past in order to relax and feel better*” was inadvertently excluded from the exhaustion subscale, resulting in a seven-item subscale and 15-item OLBI questionnaire. Scoring was reversed for negatively worded items and scores were calculated as the sum of the mean of the items in each subscale as recommended. Exhaustion was defined as a score ≥ 2.25 on the exhaustion subscale and disengagement was defined as a score ≥ 2.10 on the disengagement subscale [3]. Overall burnout, a binary variable, was defined as the presence of both exhaustion and disengagement.

### Analysis

Quantitative data were analyzed using SAS Institute, version 9.4. Descriptive statistics were conducted to provide information on participant characteristics, including the prevalence of the three OLBI measures (burnout, exhaustion and disengagement). Logistic regression was used to evaluate the association between the three OLBI measures and participant characteristics. It was decided *a priori* to adjust all models for age and gender as research shows these are known risk factors for burnout [19]. Missing data for the OLBI measures comprised 1.2% of the study sample. Since the sample size was large and missing data were minimal (< 5%), only complete participant responses were used for the analyses [20].

The association between the three OLBI measures and the intention to leave or retire earlier or later was explored using multinomial logistic regression. Responses to leave or retire either earlier or later were compared to responses to leave or retire at the same time. Responses indicating unsure or prefer not to answer were excluded from the analysis (n = 790). The multinomial logistic regression model also controlled for age and gender.

All open-text responses were analyzed for themes independently by two authors using summative content analysis in Microsoft Excel. Two authors independently reviewed the responses and categorized them into themes. Final themes were determined through an iterative discussion process between the two authors. These were validated with the larger research team that includes knowledge users. Frequency of themes were calculated.

### Ethics

The study was approved by Public Health Ontario’s Ethics Review Board (#2022 − 024.01). Participants were requested to provide informed consent before beginning the survey.

### Funding

This study received financial support from the Canadian Public Health Association.

## Results

**Fig. 1:**
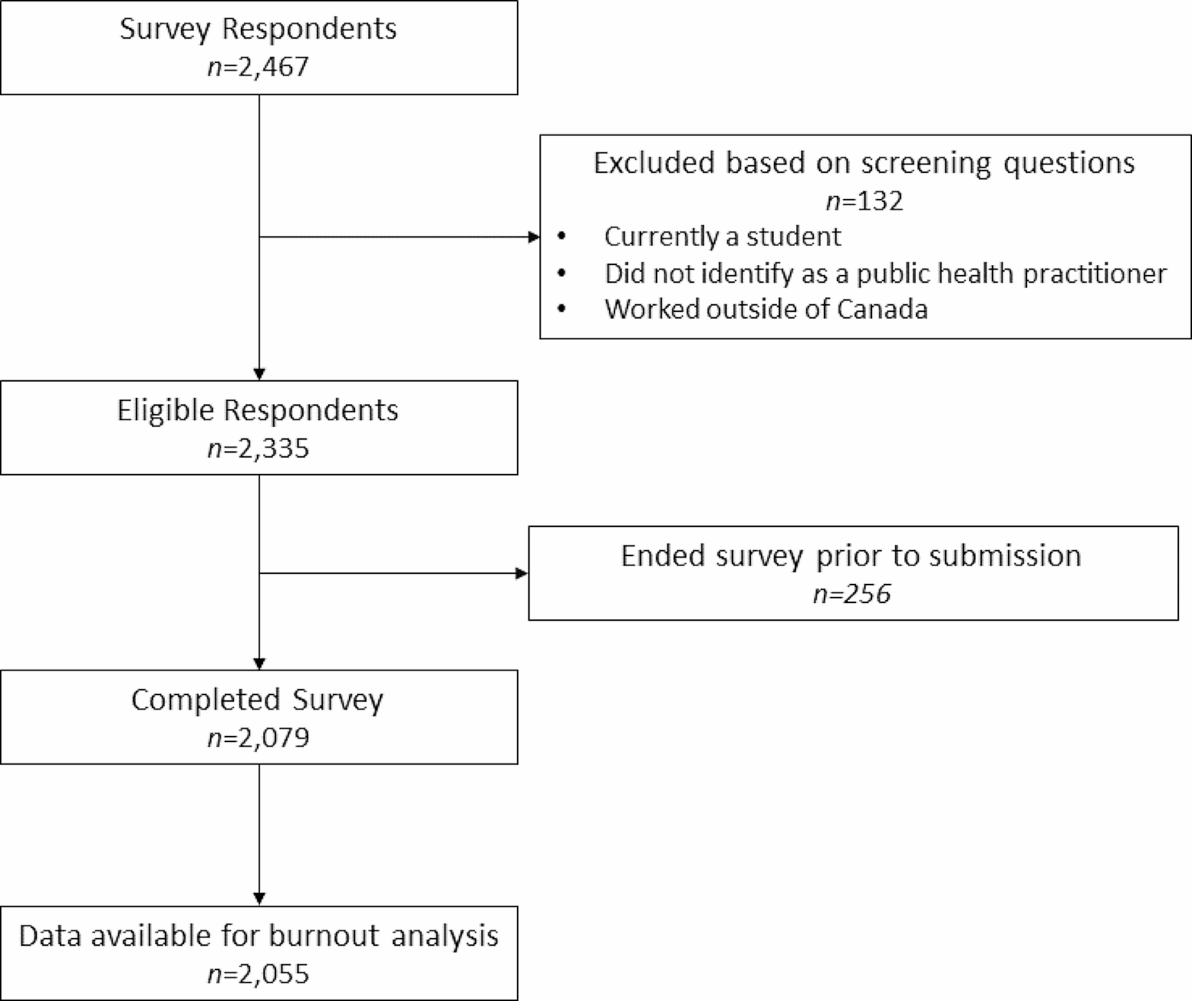
Flow diagram of study participants. OLBI: Oldenburg Burnout Inventory

Within the 8-week recruitment period between December 2022 to January 2023, 2,467 respondents consented to participate in the online survey. A total of 2,079 respondents met the survey eligibility and completed the survey. Of that, 2,055 participants completed the OLBI questions used for inferential analyses (98.8% of eligible participants) (Fig. 1). Additionally, 1,599 participants provided open-text responses to optional open-text questions (76.9% of eligible participants).

### Participant characteristics

Table 1 presents the sociodemographic and work characteristics of participants. The majority were women (87.2%), between 30 and 59 years (80.6%) and had completed a bachelor’s degree or higher (88.2%). In addition, 69.8% were married or in common law partnership, with 43% providing care to at least one child. 17.7% of respondents identified as a racialized person or person of colour.

**Table 1:** Sociodemographic and work-related characteristics of survey participants (N = 2,079)

Characteristics	n	%
**Age (years)**		
20–29	236	11.4%
30–39	551	26.5%
40–49	638	30.7%
50–59	487	23.4%
60–64	105	5.1%
≥ 65	30	1.4%
Prefer not to answer	32	1.5%
**Gender**		
Man	208	10.0%
Woman	1813	87.2%
Other (including non- binary)	16	0.8%
Prefer not to answer	42	2.0%
**Racialized Person or Person of Colour**		
Yes	367	17.7%
No	1638	78.8%
Prefer not to answer	74	3.6%
**Highest Education**		
High school diploma	23	1.1%
College diploma	199	9.6%
Bachelor’s Degree	1030	49.5%
Master’s Degree	641	30.8%
PhD and/or Professional degree (MD, DVM, DDS)	164	7.9%
Prefer not to answer	22	1.1%
**Household Income (CDN)**		
$0 to $49,999	44	2.1%
$50,000 to $99,999	568	27.3%
$100,000 to $149,999	482	23.2%
$150,000 to $199,999	400	19.2%
≥$200,000	329	15.8%
Prefer not to answer	256	12.3%
**Marital Status**		
Single, never married	375	18.0%
Married or common law	1452	69.8%
Separated or divorced	171	8.2%
Widowed	11	0.5%
Prefer not to answer	70	3.4%
**Caregiver for an Adult**		
Yes	366	17.6%
No	1673	80.5%
Prefer not to answer	40	1.9%
**Caregiver for Children < 18 years**		
Yes	894	43.0%
No	1155	55.6%
Prefer not to answer	30	1.4%
**Province or Territory**		
Alberta	102	4.9%
British Columbia	118	5.7%
Manitoba	27	1.3%
New Brunswick	7	0.3%
Newfoundland and Labrador	42	2.0%
Nova Scotia	12	0.6%
Ontario	1579	75.9%
Prince Edward Island	19	0.9%
Quebec	127	6.1%
Saskatchewan	15	0.7%
Nunavut & Northwest Territories	19	0.9%
Yukon	9	0.4%
More than one Province and/or Territory	3	0.1%
**Regional Setting**		
Urban	1071	51.5%
Rural	312	15.0%
Suburban	551	26.5%
Other	145	7.0%
**Role or Position**		
Administrative Assistant	101	4.9%
Front-line public health/ community provider	1287	61.9%
Senior management/ administration	111	5.3%
(Chief) Medical Officer of Health	22	1.1%
Associate (Chief) Medical Officer of Health	45	2.2%
Technical Expert	221	10.6%
Other (including Faculty)	267	12.8%
Prefer not to answer	25	1.2%
**Employment Status**		
Employed Full-time	1816	87.3%
Employed Part-time	169	8.1%
Casual	29	1.4%
On temporary leave	5	0.2%
Other	54	2.6%
Prefer not to answer	6	0.3%
**Employment Classification**		
Permanent	1695	81.5%
Contract	371	17.8%
Prefer not to answer	13	0.6%
**Started Working as a Public Health Practitioner**		
Before December 2019	1595	76.7%
Between December 2019 to January 2022	423	20.3%
After January 2022	61	2.9%
**Years of Work Experience**		
Less than 1 year	51	2.5%
1 to 2 years	318	15.3%
3 to 4 years	217	10.4%
5 to 9 years	341	16.4%
10 to 19 years	660	31.7%
20 to 29 years	378	18.2%
30 years or more	108	5.2%
Prefer not to answer	6	0.3%
**Work Setting**		
In office, clinic or community setting	701	33.7%
Virtual	550	26.5%
Hybrid - both virtual and in-person settings	818	39.3%
Prefer not to answer	10	0.5%
**Supported COVID-19 work-related activities**		
Yes	2026	97.5%
No	53	2.5%
**Redeployed to COVID-19 positions**		
Yes	1350	64.9%
No	711	34.2%
Prefer Not to Answer	18	0.9%
**Length of Redeployment**		
Less than 3 months	84	4.0%
3 to less than 12 months	284	13.7%
12–24 months	450	21.6%
More than 24 months	524	25.2%
Prefer not to answer	8	0.4%
Not Applicable	729	35.1%
**Threatened, Assaulted or Bullied**		
Yes	1020	49.1%
No	996	47.9%
Prefer not to answer	63	3.0%
**Felt Safe at Workplace**		
Yes	1365	65.7%
No	587	28.2%
Prefer not to answer	127	6.1%
**Workplace supports**		
Yes	1276	61.4%
No	640	30.8%
Prefer not to answer	163	7.8%
**Intention to leave or retire**		
Earlier than anticipated	371	17.8%
Same time as anticipated	390	18.8%
Later than anticipated	38	1.8%
Unsure	466	22.4%
Prefer not to answer	32	1.5%
Not applicable	782	37.6%

Regarding professional roles, 61.9% identified as front-line public health or community providers (e.g., nurse, public health inspector), 12.8% as other, 10.6% as technical experts (e.g., epidemiologist, analyst, program specialist), 5.3% as senior management/ administration, 4.9% as an administrative assistant, 3.3% as a medical officer of health (including chief or associate) and 1.2% preferred not to answer. In total, 87.3% were employed full-time and 81.5% held permanent positions. About three-quarters (76.7%) had worked in public health prior to the pandemic (i.e. since before December 2019 and 55.1% had 10 or more years of experience. Workplace arrangements varied with 33.7% working in-office or a community-based setting, 39.3% working in a hybrid model (mix of in-office and remote) and 26.5% working remotely (work-from-home). The survey was distributed to networks across Canada, and participants were from Ontario (75.9%), Quebec (6.1%), British Columbia (5.7%), Alberta (4.9%), Newfoundland and Labrador (2.0%), Manitoba (1.3%), Prince Edward Island (0.9%), Nunavut and Northwest Territories (0.9%), Saskatchewan (0.7%), Nova Scotia (0.6%), Yukon (0.4%), New Brunswick (0.3%) and more than one province and/or territory (0.1%).

During the pandemic, 97.5% of participants supported COVID-19 work-related activities and 64.9% had been redeployed to roles that directly supported the pandemic response. At the time of the survey, 46.8% had been redeployed for one or more years. A total of 49.1% of participants reported being threatened, assaulted or bullied because of their work during the pandemic. When asked about workplace safety, 65.7% of participants felt safe, 28.2% did not feel safe and 6.1% preferred not to answer. Overall, 61.4% of participants surveyed indicated that their workplace offered physical and/or mental wellbeing supports.

### Burnout

The overall prevalence of burnout was 78.7%. When the subscales were evaluated separately, 85.2% met the criteria for exhaustion and 87.7% met the criteria for disengagement (Table 2).

**Table 2:** BURNOUT, EXHAUSTION and DISENGAGEMENT prevalence and mean scores (n = 2,055)

OLBI Outcomes	Survey Participants						
	Yes		No		Mean	95% CI	
	*n*	%	*n*	%			
Burnout	1617	78.7	438	21.3	2.67	2.65	2.69
Exhaustion	1750	85.2	305	14.8	2.74	2.72	2.76
Disengagement	1782	87.7	273	13.3	2.61	2.59	2.63

OLBI: Oldenburg Burnout Inventory; CI: Confidence Interval

Results from the logistic regression analysis exploring the association between burnout measures and participant characteristics are presented in Table 3. No significant differences were observed between genders for burnout; however, the odds of exhaustion for women was 1.56 (95%CI: 1.09 to 2.25) compared to men. When adjusting for age and gender, years of experience was associated with a high level of burnout, where participants with five or more years of work experience had significantly greater odds of burnout compared to those with less than two years of experience. The adjusted odds of burnout for participants with five to nine years of work experience was 2.59 (95%CI: 1.76 to 3.83), 10 to 19 years was 2.45 (95%CI: 1.72 to 3.49), 20 to 29 years was 2.14 (95%CI: 1.42 to 3.23) and 30 or more years was 2.13 (95%CI: 1.20 to 3.81) compared to those with less than two years of work experience. There were no statistically significant differences by role/position (data not shown).

**Table 3:** Sociodemographic and work characteristics associated with odds of burnout, disengagement and exhaustion, logistic regression models adjusted for age and gender (n = 2,055)

Characteristics	BURNOUT			EXHAUSTION			DISENGAGEMENT		
	AOR	95% CI		AOR	95% CI		AOR	95% CI	
Age (years)									
20–29	Ref			Ref			Ref		
30–39	**1.60**	**1.12**	**2.29**	**1.83**	**1.22**	**2.75**	1.40	0.91	2.16
40–49	**1.72**	**1.21**	**2.45**	**1.68**	**1.14**	**2.48**	**1.59**	**1.04**	**2.45**
50–59	**1.46**	**1.02**	**2.10**	**1.65**	**1.10**	**2.49**	1.27	0.82	1.96
60+	0.92	0.58	1.47	1.02	0.60	1.72	0.68	0.40	1.16
Gender*									
Man	Ref			Ref			Ref		
Woman	1.28	0.92	1.79	**1.56**	**1.09**	**2.25**	1.15	1.15	0.76
Work Experience (years)									
≤ 2	Ref			Ref			Ref		
3 to 4	1.39	0.95	2.04	1.17	0.77	1.79	1.40	0.88	2.23
5 to 9	**2.59**	**1.76**	**3.83**	**2.40**	**1.52**	**3.78**	**2.59**	**1.60**	**4.20**
10 to 19	**2.45**	**1.72**	**3.49**	**2.31**	**1.54**	**3.46**	**2.05**	**1.36**	**3.11**
20 to 29	**2.14**	**1.42**	**3.23**	**1.96**	**1.23**	**3.13**	**1.84**	**1.13**	**2.98**
≥ 30	**2.13**	**1.20**	**3.81**	**2.08**	**1.06**	**4.08**	**2.04**	**1.03**	**4.02**
Redeployed									
No	Ref			Ref			Ref		
Yes	**1.71**	**1.37**	**2.14**	**1.51**	**1.17**	**1.95**	**1.87**	**1.44**	**2.44**
Threatened, Assaulted or Bullied									
No	Ref			Ref			Ref		
Yes	**1.82**	**1.46**	**2.27**	**1.81**	**1.40**	**2.33**	**1.97**	**1.50**	**2.58**
Felt Safe at Workplace									
Yes	Ref			Ref			Ref		
No	**2.43**	**1.84**	**3.21**	**2.77**	**1.96**	**3.89**	**2.20**	**1.57**	**3.09**
Workplace supports for physical and/or mental wellbeing									
Yes	Ref			Ref			Ref		
No	**2.26**	**1.73**	**2.95**	**1.95**	**1.44**	**2.65**	**2.55**	**1.81**	**3.59**

AOR: Adjusted Odds Ratio; CI: Confidence Interval

*Results for non-binary and other gender responses not shown due to small sample size

The adjusted odds of burnout for participants redeployed to the pandemic response was 1.71 (95%CI: 1.37 to 2.14) times that of participants not redeployed. In addition, the adjusted odds of burnout were greater for participants who were threatened, assaulted or bullied during the pandemic compared to those who were not (AOR = 1.82; 95%CI: 1.46 to 2.27) and for those who did not feel safe in the workplace compared to those that did (AOR = 2.43; 95%CI 1.84 to 3.21). Workplace supports for physical and mental wellbeing were observed to be significantly associated with burnout. The adjusted odds of burnout for participants not offered workplace supports was 2.26 (95%CI: 1.73 to 2.95) times that for those offered supports. Similar associations were observed for the exhaustion and disengagement subscales, see Table 3.

### Intention to leave or retire

Table 4 shows the results of a multinomial logistic regression analysis between burnout measures and intention to leave or retire as a result of the COVID-19 pandemic. The odds of intending to leave or retire earlier than anticipated compared to leaving or retiring at the same time for participants with burnout was 6.13 (95%CI: 3.71 to 10.13) times that of those without burnout when adjusting for age and gender. Similar associations were observed for participants with exhaustion and disengagement in relation to their intention to leave or retire early. Those with burnout had decreasing odds of leaving or retiring later, compared to leaving or retiring at the same time, than those without burnout (AOR = 0.46; 95%CI: 0.22 to 0.96).

**Table 4:** Association between burnout and intention to leave or retire earlier or later than previously anticipated, compared to no change in plans to leave or retire (i.e., same time). Multinomial logistic regression model adjusted for age and gender. (n = 1265, 466 with unsure retirement plans excluded)

	Leave or Retire Earlier				Leave or Retire Later		
	AOR	95% CI			AOR	95% CI	
Burnout							
No	Ref				Ref		
Yes	**6.13**	**3.71**	**10.13**		**0.46**	**0.22**	**0.96**
Exhaustion							
No	Ref				Ref		
Yes	**7.01**	**3.71**	**13.23**		**0.41**	**0.18**	**0.90**
Disengagement							
No	Ref				Ref		
Yes	**6.45**	**3.41**	**12.21**		0.64	0.29	1.44

AOR: Adjusted Odds Ratio; CI: Confidence Interval

### Summary of open-text responses

A total of 1,599 participants (76.9% of eligible participants) provided free-text responses to open-ended questions specific to harassment, workplace safety and supports, and motivations for working during a pandemic (Table 5).

**Table 5:** Themes from open-text responses (n = 1,599)

Theme	Category
**1. Forms of Harassment**	● Common types: name calling, yelling, and general rudeness. ● Other types: hate letters, death threats, gun violence, animal attacks, threat of lawsuits, unauthorized surveillance or recording, spat-on, having objects thrown, barricaded or locked-down at workplace from protestors.
**Reasons for Harassment**	● Public health mandates including vaccination mandates, travel restrictions, mask mandates, temporary school or business closures, quarantine and contact tracing measures during the pandemic. ● Lack of vaccine availability: difficulties booking appointments, availability of vaccines and preferred vaccine product type.
**Barriers to workplace safety**	● Harassment from the public ● Lack support from management and/or workplace bullying ● Poor training and increased workload ● Risk of COVID-19 exposure at the workplace from lack of social distancing or crowded spaces, poor ventilation and hygiene practices ● Contact with COVID-19 positive staff ● Inaccessibility to vaccines early on
**Facilitators to workplace safety**	● Remote work arrangements (e.g., work-from-home) ● Support from colleagues or management ● Clear and consistent communication within the workplace ● Increased security measures at workplace ● Adequate supply of personal protective equipment (PPE) ● Increased cleaning/disinfecting measures ● Social distancing
**Types of workplace supports offered by employers**	● Employee and Family Assistance Program (EFAP) ● Mental health programs (e.g., virtual coffee breaks, mental health webinars, apps or e-mental health tools, counseling services) ● Wellness programs ● Informal supports by colleagues or management ● Increased workplace benefits including additional paid sick leave ● Remote work arrangements ● Measures to prevent infections (e.g., PPE and hand sanitizer, onsite vaccinations, COVID-19 tests) ● Frequent check-ins and/or reassurance by management ● Staff appreciation events
**Motivating factors to continue working during the pandemic**	● Engaging with colleagues ● Social support networks (family and friends) ● Remote work arrangements ● Feeling that work was rewarding, impactful and significant ● Positive community feedback ● Faith and faith groups ● Compensation and job security

### Forms of harassment

A total of 1,504 participants completed open-text responses on forms of harassment and reasons for harassment. The majority of threats, assaults or bullying came from clients and patients, in the form of name calling, yelling or general rudeness. Less common forms of harassment included hate letters, death threats, and having objects thrown.

### Reasons for harassment

Participants commonly reported feeling harassed because of public health mandates and lack of vaccine availability. Descriptions of public health mandates included vaccination mandates, travel restrictions, mask mandates, and temporary school or business closures. Lack of vaccine availability descriptions included difficulty booking appointments and lack of preferred vaccine type.

### Barriers to workplace safety

A total of 553 participants provided open-text comments on barriers to workplace safety. Harassment from the public was the main reason participants identified for not feeling safe at the workplace. Other barriers to workplace safety described were lack of support from management, increased workload, poor training for new duties and roles, and risk of COVID-19 exposure or infection.

### Facilitators to workplace safety

A total of 730 participants provided open-text responses describing facilitators to workplace safety. Many participants felt that work-from-home arrangements improved their workplace safety. Additionally support from colleagues or management, clear and consistent messaging, and increased safety measures through security or infection prevention and control contributed to feelings of workplace safety.

### Types of workplace supports offered by employers

A total of 1,065 participants described workplace supports offered by employers. Overall, there were mixed reactions towards wellness supports being offered at the workplace. Some found them helpful and were appreciative, while others felt they were insufficient or did not have time for them. The most common type of workplace support described were Employee and Family Assistance Programs.

### Motivating factors to continue working during the pandemic

A total of 1,465 respondents described motivating factors to continue working during the pandemic. Many respondents attributed engaging with colleagues, social networks (family and friends), remote work arrangements, and the rewarding and impactful nature of their work as factors that kept them motivated during the pandemic.

## Discussion

This cross-sectional study is one of the first to explore the prevalence of burnout among the Canadian public health workforce. Results showed that three years into the pandemic, burnout was extremely common, affecting 78.7% of respondents. Moreover, participants suffered from high rates of exhaustion (85.2%) and disengagement (87.7%). These levels of burnout are in the higher range among other public health workforces globally [11–14]. This was not surprising given the immense burden associated with managing the long-term response to the COVID-19 pandemic professionally and personally. These findings further compound previously identified public health system challenges that may be contributing to chronic work-related stressors facing the public health workforce. Prior to the pandemic, many public health agencies in jurisdictions across Canada were severely understaffed and underfunded, placing a great deal of pressure on a resource-limited workforce to maintain essential public health services [21]. The pandemic may have exacerbated these pressures given the “all-hands-on-deck” approach, resulting in longer working hours, less ability to take vacation and little to no capacity to maintain essential services. As public health workers begin to catch up on neglected services, there may be little respite from the stresses associated with high work demands.

The pandemic saw an extraordinary increase in the level of harassment and personal attacks on health workers during COVID-19 [22, 23]. In the US, personal attacks against health officers have been well documented [24]. In fact, survey data showed an increase in US adults that felt justified in harassing or threatening public health workers because of mandates and “pandemic fatigue” [25]. Our study showed that the Canadian public health workforce was not immune to this phenomenon with 49.1% of participants reporting COVID-19-related bullying, threats or assaults mainly over mandates or lack of vaccine availability. We also found that COVID-19-related discrimination was associated with higher odds of burnout. Similarly, another study observed that COVID-19-related discrimination was associated with depressive symptoms and suicidal ideation among healthcare providers [26]. Future efforts to safeguard public health workers may require different strategies, such as tailored outreach to groups negatively impacted by public health mandates, increased security at workplaces, trauma support networks, incident harassment reporting systems, and others [23].

Improving workplace safety is an important area for further study as we found that public health workers who felt unsafe had a higher odds of burnout. Although not directly measured, in the open-text portion of the survey, participants discussed important barriers to their safety including the risk of COVD-19 infection, especially early on when vaccines were not available, harassment, and poor workplace hygiene practices. This aligns with research findings that, during the pandemic, front-line healthcare workers experienced high levels of stress, safety-related fear, worry, anxiety and exhaustion [27]. In contrast, participants discussed remote work arrangements, increased hygiene protocols, and security measures as factors that improved their workplace safety. A systematic review and meta-analysis of controlled interventions for burnout in physicians found that these were associated with small significant reductions in burnout scores and that these would be boosted by the use of organization-level approaches [28]. A further systematic review of interventions for frontline health and social care professionals during and after a disease outbreak, epidemic or pandemic found a lack of evidence [29]. The small body of literature in this area supports the need for research on effective interventions at the individual, organizational and public health system levels, as well as the importance of evaluation of interventions currently in use.

This survey also gave us insight into how burnout is associated with the career-planning of public health workers. We found that the intention to leave or retire early was highly associated with burnout and its subscales of exhaustion and disengagement. These findings are consistent with healthcare studies that examined the link between burnout and intention to leave among nurses and physicians in Canada [30, 31]. Although both exhaustion and disengagement are important predictors of professional turnover, exhaustion is thought to be more salient of a predictor among healthcare professionals as it is believed to indirectly affect their professional commitment [31]. This may explain our observation that exhaustion was associated with decreased odds of intention to leave or retire later. The public health workforce is an ageing population facing an exodus of scheduled retirements [32]. The potential additional turnover of workers from burnout may have serious consequences to the public health system’s ability to effectively provide essential services and respond to future emerging threats.

There were several limitations to this study. Given its cross-sectional design, findings may not capture the peak and fluctuations in burnout levels during the pandemic. In addition, this study cannot conclude any causal relationships. Distribution of the survey through public health associations and networks may have introduced selection bias as individuals with burnout who left or retired prior to the release of our questionnaire would not be included. This may have led to an underestimation of the burnout prevalence. However, those with greater level of burnout may have been more likely to respond to this survey resulting in an overestimation of the burnout prevalence. All measures were self-reported and susceptible to response bias. The majority of participants were from Ontario which may limit the generalizability to other provinces and territories. However, considering the high levels of burnout that have been reported globally and among multiple health workforces, we believe these findings may be applicable to other jurisdictions. The strengths of the study included the large sample size and inclusive definition of a diverse public health workforce.

## Conclusion

After three years of the COVID-19 pandemic, it is evident that the Canadian public health workforce is facing high levels of burnout. Our results showed that work-related factors including COVID-19 redeployment, increased work experience, and harassment were all associated with higher odds of burnout and that burnout was associated with greater odds of intending to leave or retire early. Public health organizations should consider the importance of workplace supports and workplace safety in potentially mitigating burnout. Future studies should seek to systematically enumerate and understand the public health workforce, including the evolution of burnout and recovery over time, along with identifying effective interventions to prevent, mitigate and recover from burnout at the system, organization and individual levels.

## Electronic supplementary material

Below is the link to the electronic supplementary material.

Supplementary Material 1

## Data Availability

The datasets generated and analysed during the current study are not publicly available due to privacy but are available from the corresponding author on reasonable request. Code used to analyse the data is available by request.

## Abbreviations

AOR: Adjusted odds ratio
CHERRIES: Checklist for Reporting Results of Internet E-Surveys
CI: Confidence interval
COVID-19: Coronavirus disease 2019
OLBI: Oldenburg Burnout Inventory
OR: Odds ratio
STROBE: Strengthening the Reporting of Observational Studies in Epidemiology
USA: United States of America

## References

[1] Public Health Agency of Canada. COVID-19 epidemiology update: Summary [Internet]. Ottawa: Public Health Agency of Canada; 2023 [cited 2023 Jul 28]. Available from: https://health-infobase.canada.ca/covid-19/.

[2] Public Health Agency of Canada. COVID-19: Canada’s response [Internet]. Ottawa: Public Health Agency of Canada; 2023 [cited 2023 Jun 12]. Available from: https://www.canada.ca/en/public-health/services/diseases/2019-novel-coronavirus-infection/canadas-reponse.html.

[3] Demerouti E, Bakker AB, Nachreiner F, Schaufeli WB. The job demands-resources model of burnout. J Appl Psychol. 2001;86(3):499–512. 11419809

[4] Menon NK, Shanafelt TD, Sinsky CA, Linzer M, Carlasare L, Brady KJS, et al. Association of Physician Burnout with suicidal ideation and medical errors. JAMA Netw Open. 2020;3(12):1–14. 10.1001/jamanetworkopen.2020.28780 PMC7726631 33295977

[5] de Wit K, Mercuri M, Wallner C, Clayton N, Archambault P, Ritchie K, et al. Canadian emergency physician psychological distress and burnout during the first 10 weeks of COVID-19: a mixed‐methods study. J Am Coll Emerg Physicians Open. 2020;1(5):1030–8. 32905025 10.1002/emp2.12225 PMC7461319

[6] Salvagioni DAJ, Melanda FN, Mesas AE, González AD, Gabani FL, de Andrade SM. Physical, psychological and occupational consequences of job burnout: a systematic review of prospective studies. PLoS ONE. 2017;12(10):e0185781. 28977041 10.1371/journal.pone.0185781 PMC5627926

[7] Canadian Medical Association. CMA National physician health survey: A national snapshot [Internet]. Canadian Medical Association; 2018 [cited 2023 July 28]. Available from: https://www.cma.ca/cma-national-physician-health-survey-national-snapshot.

[8] Khan N, Palepu A, Dodek P, Salmon A, Leitch H, Ruzycki S, et al. Cross-sectional survey on physician burnout during the COVID-19 pandemic in Vancouver, Canada: the role of gender, ethnicity and sexual orientation. BMJ Open. 2021;11(5):e050380. 33972345 10.1136/bmjopen-2021-050380 PMC8111871

[9] Singh S, Farrelly A, Chan C, Nicholls B, Nazeri-Rad N, Bellicoso D, et al. Prevalence and workplace drivers of Burnout in Cancer Care Physicians in Ontario, Canada. JCO Oncol Pract. 2022;18(1):e60–71. 34506217 10.1200/OP.21.00170

[10] Registered Nurses Association of Ontario. Nursing Through Crisis: A Comparative Perspective [Internet]. Registered Nurses Association of Ontario 2022 [cited 2023 July 28]. Available from: https://rnao.ca/sites/default/files/2022-05/Nursing%20Through%20Crisis%20-%20A%20Comparative%20Analysis%202022.pdf.

[11] Ibrahim F, Samsudin EZ, Chen XW, Toha HR. The prevalence and Work-Related Factors of Burnout among Public Health Workforce during the COVID-19 pandemic. J Occup Environ Med. 2022;64(1):E20–7. 34789681 10.1097/JOM.0000000000002428 PMC8715934

[12] Stone KW, Kintziger KW, Jagger MA, Horney JA. Public health workforce burnout in the covid-19 response in the U.S. Int J Environ Res Public Health. 2021;18(8). 10.3390/ijerph18084369 PMC8074254 33924084

[13] Lee J, Jang S-N, Kim N-S. Burnout Among Public Health Workers During the COVID-19 Pandemic in South Korea. Journal of Occupational and Environmental Medicine [Internet]. 2023;65(3). Available from: https://journals.lww.com/joem/Fulltext/2023/03000/Burnout_Among_Public_Health_Workers_During_the.19.aspx. 10.1097/JOM.0000000000002773 PMC9987640 36728934

[14] Lu S, Zhang L, Klazinga N, Kringos D. More public health service providers are experiencing job burnout than clinical care providers in primary care facilities in China. Human Resources for Health [Internet]. 2020;18(1):1–11. 10.1186/s12960-020-00538-z. 10.1186/s12960-020-00538-z PMC7711271 33272284

[15] Eysenbach G. Improving the quality of web surveys: the Checklist for reporting results of internet E-Surveys (CHERRIES). J Med Internet Res. 2004;6(3):1–6. 10.2196/jmir.6.3.e34 PMC1550605 15471760

[16] Elm Ev, Altman DG, Egger M, Pocock SJ, Gøtzsche PC, Vandenbroucke JP. The strengthening the reporting of Observational studies in Epidemiology (STROBE) statement: guidelines for reporting observational studies. J Clin Epidemiol. 2008;61(4):344–9. 18313558 10.1016/j.jclinepi.2007.11.008

[17] Butler-Jones D. The Chief Public Health Officer’s report on the state of public health in Canada: Addressing health inequalities [Internet]. 2008 [cited 2023 July 28]. Available from: https://www.canada.ca/content/dam/phac-aspc/migration/phac-aspc/cphorsphc-respcacsp/2008/fr-rc/pdf/CPHO-Report-e.pdf.

[18] Halbesleben JRB, Demerouti E. The construct validity of an alternative measure of burnout: investigating the English translation of the Oldenburg Burnout Inventory. Work Stress. 2005;19(3):208–20.

[19] Marchand A, Blanc ME, Beauregard N. Do age and gender contribute to workers’ burnout symptoms? Occup Med. 2018;68(6):405–11. 10.1093/occmed/kqy088 PMC6093338 29912439

[20] Jakobsen JC, Gluud C, Wetterslev J, Winkel P. When and how should multiple imputation be used for handling missing data in randomised clinical trials - a practical guide with flowcharts. BMC Med Res Methodol. 2017;17(1):1–10. 29207961 10.1186/s12874-017-0442-1 PMC5717805

[21] Guyon A, Perreault R. Public health systems under attack in Canada: Evidence on public health system performance challenges arbitrary reform. Canadian Journal of Public Health [Internet]. 2016;107(3):e326–9. 10.17269/CJPH.107.5273. 10.17269/CJPH.107.5273 PMC6972346 27763850

[22] Dye TD, Alcantara L, Siddiqi S, Barbosu M, Sharma S, Panko T, et al. Risk of COVID-19-related bullying, Harassment and stigma among healthcare workers: an analytical cross-sectional global study. BMJ Open. 2020;10(12):1–15. 10.1136/bmjopen-2020-046620 PMC7780430 33380488

[23] Ward JA, Stone EM, Mui P, Resnick B. Pandemic-related Workplace Violence and its impact on Public Health officials, March 2020–January 2021. Am J Public Health. 2022;112(5):736–46. 35298237 10.2105/AJPH.2021.306649 PMC9010912

[24] Mello MM, Greene JA, Sharfstein JM. Attacks on Public Health officials during COVID-19. JAMA. 2020;324(8):741–2. 32777019 10.1001/jama.2020.14423

[25] Topazian RJ, McGinty EE, Han H, Levine AS, Anderson KE, Presskreischer R, et al. US adults’ beliefs about harassing or threatening Public Health officials during the COVID-19 pandemic. JAMA Netw Open. 2022;5(7):E2223491. 35904784 10.1001/jamanetworkopen.2022.23491 PMC9338413

[26] Campo-Arias A, Jiménez-Villamizar MP, Caballero-Domínguez CC. Healthcare workers’ distress and perceived discrimination related to COVID-19 in Colombia. Nurs Health Sci. 2021;23(3):763–7. 33999491 10.1111/nhs.12854 PMC8242481

[27] Subramony M, Golubovskaya M, Keating B, Solnet D, Field J, Witheriff M. The influence of pandemic-related workplace safety practices on frontline service employee wellbeing outcomes. J Bus Res. 2022;149:363–74. 35637699 10.1016/j.jbusres.2022.05.040 PMC9132582

[28] Panagioti M, Panagopoulou E, Bower P. Controlled interventions to reduce burnout in physicians: a systematic review and meta-analysis. JAMA Intern Med. 2017;177(2):195–205. 27918798 10.1001/jamainternmed.2016.7674

[29] Pollock A, Campbell P, Cheyne J, Cowie J, Davis B, McCallum J, McGill K, et al. Interventions to support the resilience and mental health of frontline health and social care professionals during and after a Disease outbreak, epidemiic or pandemic: a mixed methods systematic review. Cochrane Database Systematic Reviews. 2020;11(11):CD013779. 10.1002/14651858.CD013779 PMC8226433 33150970

[30] Chênevert D, Kilroy S, Johnson K, Fournier P. The determinants of burnout and professional turnover intentions among Canadian physicians : application of the job demands-resources model. BMC Health Serv Res. 2021;21(1):1–10. 34544396 10.1186/s12913-021-06981-5 PMC8454159

[31] Jourdain G, Chênevert D. Job demands-resources, burnout and intention to leave the nursing profession: a questionnaire survey. Int J Nurs Stud. 2010;47(6):709–22. 20138278 10.1016/j.ijnurstu.2009.11.007

[32] Leider JP, Coronado F, Beck AJ, Harper E. Reconciling supply and Demand for State and Local Public Health Staff in an era of retiring Baby boomers. Am J Prev Med. 2018;54(3):334–40. 29336862 10.1016/j.amepre.2017.10.026 PMC6944191

