# Supplementary material for: Burnout among public health workers in Canada: a cross-sectional study
Source: BMC Public Health. 2024 Jan 2;24:48. doi: 10.1186/s12889-023-17572-w (PMC10763416; doi:10.1186/s12889-023-17572-w)
Supplement: Supplementary file 1 — Supplementary Material 1 [file 12889_2023_17572_MOESM1_ESM.docx]

# APPENDICES

**Appendix A – Canadian Public Health Associations and Professional Groups who distributed the invitation to participate in the survey**

- Association of Local Public Health Agencies (alPHa)
- Association of Public Health Epidemiologists in Ontario (APHEO)
- Canadian Vaccination Evidence Resource and Exchange Centre (CANVax)
- Canadian Institute of Public Health Inspectors (CIPHI)
- Canadian Public Health Association (CPHA)
- Immunize Canada
- Institute national de santé publique du Québec (INSPQ)
- National Collaborating Centre for Methods and Tools (NCCMT)
- Provincial public health associations
- Ontario Public Health Chief Nursing Officers
- Public Health Physicians of Canada (PHPC)
- Urban Public Health Network (UPHN)
- Community Health Nurses of Canada
- Ontario Council of Medical Officers of Health (COMOH)
- Public Health Ontario (PHO) Connect

Appendix B - Public Health Workforce Burnout Survey

**Work Characteristics**

1. Do you identify as a public health practitioner in Canada?

A public health practitioner is someone whose work aligns with one or more of the core functions for Public Health in Canada:

- Population health assessment
- Health protection
- Health surveillance
- Disease and injury prevention
- Health promotion
- Emergency preparedness and response

- Yes
- No (if selected end survey)

1. Are you currently a student doing a practicum with Public Health
   - Yes (if selected end survey)
   - No
2. Do you currently work in a public health capacity?

- Yes
- No

1. When did you start working as a public health practitioner?
   - Before December 2019
   - Between December 2019 to January 2022
   - After January 2022
2. In which province or territory do you currently work?
   - Alberta
   - British Columbia
   - Manitoba
   - New Brunswick
   - Newfoundland and Labrador
   - Nova Scotia
   - Ontario
   - Prince Edward Island
   - Quebec
   - Saskatchewan
   - Nunavut
   - Northwest Territories
   - Yukon
   - More than one Province and/or Territory
   - I work outside of Canada (if selected end survey)
3. Which of the following best describes your primary work setting during the pandemic? Check all that apply.

- Federal Government
- Provincial/territorial government
- Municipal/regional government
- Non-Governmental Organization
- Other. Please specify: __________________
- Prefer not to answer

1. What best describes your current region of work setting?
   - Urban (in the city)
   - Rural (in the country)
   - Suburban (mixed-use or residential area, existing either as part of a city area or as a separate residential community within commuting distance of a city).
   - Other. Please Specify:___________
2. At any point between 2019 and present, have you supported COVID-19 work-related activities?
   - Yes
   - No
3. What was your usual employment status during the pandemic?
   - Employed Full-time
   - Employed Part-time
   - Casual
   - On temporary leave
   - Not working - quit
   - Not working - retired
   - Other
   - Prefer not to answer
4. How would you classify your position?

- Permanent
- Contract
- Prefer not to answer

1. How many years of experience do you have working in public health?
   - Less than 1 year
   - 1 to 2 years
   - 3 to4 years
   - 5 to 9 years
   - 10 to 19 years
   - 20 to 29 years
   - 30 years or more
   - Prefer not to answer
2. Is your primary position considered: Check all that apply

- Administration assistant/support (e.g., Executive Assistant, Administrative Assistant, Program Assistant)
- Front-line public health/community provider (e.g., Health Promoter, Public Health Nurse, Public Health Inspector, Community Worker)
- Management/administration (e.g. Director, Executive, Manager, Supervisor)
- Chief Medical Officer of Health, Medical Officer of Health, Medical Health Officer
- Chief Medical Officer of Health, Associate Chief Medical Officer of Health, Associate Medical Health Officer
- Technical expert (e.g., Health Analyst, Advisor, Epidemiologist, Evaluator, Scientist)
- Other. Please specify:_____________
- Prefer not to answer

1. What was/is your usual work setting during the pandemic (i.e., between 2019 and present)?
   - In office, clinic or community setting
   - Virtual
   - Hybrid - both virtual and in-person settings
   - Prefer not to answer
2. Were you redeployed from your usual work at any time during the pandemic (i.e., between 2019 and present)?
   - Yes
   - No
   - Prefer not to answer
3. (If yes to 14) Approximately how long were you redeployed for?

- Less than 3 months
- 3 to less than 12 months
- 12 to 24 months
- More than 24 months
- Prefer not to answer

1. As a result of the COVID-19 pandemic, I have decided to:

- Leave or retire earlier than I previously anticipated
- Leave or retire at the same time as I previously anticipated
- Leave or retire later than I previously anticipated
- Unsure
- Prefer not to answer
- Not applicable

1. Have you been threatened, assaulted, or bullied because of your work during the pandemic?
   - Yes
   - No
   - Prefer not to answer

If yes, please describe briefly. (text box- optional question- max 50 characters)

1. Did you feel safe in your workplace during the COVID-19 pandemic?
   - Yes
   - No
   - Prefer not to answer

If Yes or No, please describe briefly. (text box- optional question – max 50 characters).

1. Did your workplace offer supports for your physical and/or mental wellbeing during COVID-19?
   - Yes
   - No
   - Prefer not to answer

If yes, please describe briefly. (text box – optional question – max 50 characters)

**Oldenburg Burnout Inventory**

|  | strongly agree | agree | disagree | strongly disagree |
| --- | --- | --- | --- | --- |
| 1. I always find new and interesting aspects in my work | 1 | 2 | 3 | 4 |
| 1. There are days when I feel tired before I arrive at work | 4 | 3 | 2 | 1 |
| 1. It happens more and more often that I talk about my work in a negative way | 4 | 3 | 2 | 1 |
| 1. I can tolerate the pressure of my work very well | 1 | 2 | 3 | 4 |
| 1. Lately, I tend to think less at work and do my job almost mechanically | 4 | 3 | 2 | 1 |
| 1. I find my work to be a positive challenge | 1 | 2 | 3 | 4 |
| 1. During my work, I often feel emotionally drained | 4 | 3 | 2 | 1 |
| 1. Over time, one can become disconnected from this type of work | 4 | 3 | 2 | 1 |
| 1. After working, I have enough energy for my leisure activities | 1 | 2 | 3 | 4 |
| 1. Sometimes I feel sickened by my work tasks | 4 | 3 | 2 | 1 |
| 1. After my work, I usually feel worn out and weary | 4 | 3 | 2 | 1 |
| 1. This is the only type of work that I can imagine myself doing | 1 | 2 | 3 | 4 |
| 1. Usually, I can manage the amount of my work | 1 | 2 | 3 | 4 |
| 1. I feel more and more engaged in my work | 1 | 2 | 3 | 4 |
| 1. When I work, I usually feel energized | 1 | 2 | 3 | 4 |

**Demographics**

1. What is your age in years?

- Under 20 years old
- 20-29 years old
- 30-39 years old
- 40-49 years old
- 50-59 years old
- 60-64 years old
- 65 years old or older
- Prefer not to answer

1. Please indicate which term best describes your gender identity:

- Woman
- Man
- Non-binary
- Other
- Prefer not to answer

1. Do you identify as a racialized person/person of color?

- Yes
- No
- Prefer not to answer

1. What is the highest level of education you have completed?

- Some high school or less
- High school diploma
- College diploma
- Bachelor’s Degree
- Master’s Degree
- Doctoral degree
- Prefer not to answer

1. What was your total household income in Canadian dollars from all sources before taxes last year?

- Less than $34,999
- $35,000 to $49,999
- $50,000 to $74,999
- $75,000 to $99,999
- $100,000 to 124,999
- $125,000 to $149,999
- $150,000 to $174,999
- $175,000 to $199,999
- More than $200,000
- Prefer not to answer

1. Are you a caregiver for any children less than 18 years of age?

- Yes
- No
- Prefer not to answer

1. Are you a caregiver for any adult dependents?

- Yes
- No
- Prefer not to answer

1. What is your current marital status?

- Single, never married
- Married or common law
- Separated or divorced
- Widowed
- Prefer not to answer

1. Are there positive things that kept you motivated while working in public health during the pandemic?

- Yes
- No
- Prefer not to answer

If yes, please describe briefly. (text box – optional question – max 50 characters)

**Debrief page**

If you are experiencing burnout, you are not alone and support is available to you. Please follow these links to find information about burnout and options for support:

- [Canadian Mental Health Association](https://www.camh.ca/en/health-info/mental-health-and-covid-19/information-for-professionals)
- [Canadian Medical Association](https://www.cma.ca/physician-wellness-hub/resources/resilience/managing-burnout-for-healthcare-providers)
- [Canadian Nurses Association](https://www.cna-aiic.ca/en/policy-advocacy/advocacy-priorities/mental-health)
- You can talk to a mental health professional via [Wellness Together Canada](https://www.wellnesstogether.ca/en-CA?lang=en-ca) by calling 1-866-585-0445 or text WELLNESS to 686868 for youth or 741741 for adults. It is free and confidential.
- Other resources may be available through your provincial/territorial government, your employer and your primary healthcare provider

Thank you so much for participating.

Would you like to enter the draw for a Visa gift card or receive a copy of the study results? If yes, you will be taken to another site to enter your name and email address. A separate site is used so that your name and email cannot be linked with your survey responses.

- - Yes – redirect to another site
  - No – survey window will close
